# Mean platelet volume, platelet distribution width and carcinoembryonic antigen to discriminate gastric cancer from gastric ulcer

**DOI:** 10.18632/oncotarget.15898

**Published:** 2017-03-04

**Authors:** Zhi-Yuan Yun, Na Li, Xin Zhang, Huan Zhang, Yue Bu, Yuxiang Sun, Tiemin Liu, Rui-Tao Wang, Kai-Jiang Yu

**Affiliations:** ^1^ Department of Internal Medicine, Harbin Medical University Cancer Hospital, Harbin Medical University, Harbin, Heilongjiang, China; ^2^ Department of Intensive Care Unit, Harbin Medical University Cancer Hospital, Harbin Medical University, Harbin, Heilongjiang, China; ^3^ Departments of Pediatrics & Molecular and Cellular Biology, Children’s Nutrition Research Center, Huffington Center on Aging, Baylor College of Medicine, Houston, TX, USA; ^4^ Department of Internal Medicine, Division of Hypothalamic Research, UT Southwestern Medical Center, Dallas, TX, USA

**Keywords:** gastric cancer, mean platelet volume, platelet distribution width, diagnosis

## Abstract

Activated platelets are involved in cancer development and progression. Mean platelet volume (MPV) and platelet distribution width (PDW) are early indexes of platelet activation. The objectives of this study were to investigate the ability of MPV, PDW and carcinoembryonic antigen (CEA) individually or in combination, to distinguish between gastric cancer and gastric ulcer. The study involved 194 patients with gastric cancer, 191 patients with gastric ulcer, and 185 control subjects. Subjects’ characteristics and hematologic tests data at initial diagnosis were collected. We found that MPV levels are significantly increased and PDW levels are significantly reduced in patients with gastric ulcer and in control subjects compared with those in gastric cancer. When the area under the curve (AUC) was used to analyze control subjects *versus* gastric cancer, the combination of PDW and CEA exhibited a significantly larger AUC of 0.939 (0.910-0.961) compared with the combination of MPV and CEA (*p* = 0.0045). When AUC was used to analyze gastric ulcer *versus* gastric cancer, PDW alone had the high specificity (98.5%) and high sensitivity (97.4%). In conclusion, combined use of MPV, PDW and CEA can accurately distinguish gastric cancer from gastric ulcer and controls. Further studies in larger samples are warranted.

## INTRODUCTION

Gastric ulcer is positively associated with the risk of developing gastric cancer [1]. Moreover, clinical symptoms of early gastric cancer could not be used to distinguish gastric cancer from ulcers. Although endoscopy test offers high diagnostic accuracy, it is inconvenient and could lead to additional complications. Therefore, additional markers to distinguish early gastric cancer from ulcers are necessary.

Activated platelets are involved in cancer progression and metastases [2, 3]. Mean platelet volume (MPV) is a marker of activated platelets and is associated with gastric cancer, ovarian cancer, lung cancer, colon cancer, and breast cancer [4-8]. Platelet distribution width (PDW), another platelet index, indicates variation in platelet size [9]. Carcinoembryonic antigen (CEA) has been widely used as a diagnostic, screening, and monitoring marker in clinical practice. However, CEA lacks high sensitivity and specificity.

Therefore, the objective of this study was to investigate the ability of MPV, PDW and CEA individually or in combination, to distinguish between gastric cancer and gastric ulcer.

## RESULTS

The characteristics of the patients with gastric cancer and gastric ulcer and control subjects are demonstrated in Table 1. One-way ANOVA analysis indicated a significant group difference in CEA, platelet count, MPV, PDW, and haemoglobin levels. However, there are no markedly difference in age, BMI, white blood cell, and the percentage of male and current smokers among three groups.

**Table 1:** Clinical and laboratory characteristics of the participants.

Variables	Controls	Gastric ulcer	Gastric cancer	*p*-value
Number	185	191	194	
Age (years)	54.6 (3.7)	54.9 (5.2)	54.7 (9.7)	0.899
Gender (male, %)	104 (56.2)	111 (58.1)	118 (60.8)	0.657
BMI (kg/m^2^)	23.5 (3.2)	23.4 (3.4)	22.9 (3.2)	0.189
Current smoker (%)	91 (49.2)	97 (50.8)	101 (52.1)	0.855
CEA (ng/ml)	0.86 (0.79–0.96)^a^	1.33 (0.90–1.76)^b^	2.09 (1.18–3.67)^c^	< 0.001
WBC (×10^9^/L)	6.6 (1.5)	6.7 (1.5)	6.3 (2.2)	0.069
Platelet (×10^9^/L)	218.5 (58.9)^a^	252.7 (58.4)	268.4 (88.9)^c^	< 0.001
MPV (fL)	10.3 (1.3)^a^	10.6 (1.3)^b^	8.7 (1.4)^c^	< 0.001
PDW (%)	14.8 (2.3)^a^	12.8 (2.2)^b^	17.4 (1.1)^c^	< 0.001
Haemoglobin (g/dl)	140.7 (13.8)^a^	134.5 (10.6)	132.7 (21.2)^c^	< 0.001

Data are presented as means (SD) or median (interquartile range) or percentage. BMI, body mass index; CEA, carcinoembryonic antigen; WBC, white blood cells; MPV, mean platelet volume; PDW, platelet distribution width. *P-*value was calculated by one-way ANOVA test or KruskaleWallis *H* test or chi-square test. A indicates a significant difference (*p* < 0.05) by comparison of controls and gastric ulcer using post hoc Tukey test or Mann-Whitney *U* test. B indicates a significant difference (*p* < 0.05) by comparison of gastric ulcer and gastric cancer using post hoc Tukey test or Mann-Whitney *U* test. C indicates a significant difference (*p* < 0.05) by comparison of controls and gastric cancer using post hoc Tukey test or Mann-Whitney *U* test.

**Figure 1:**
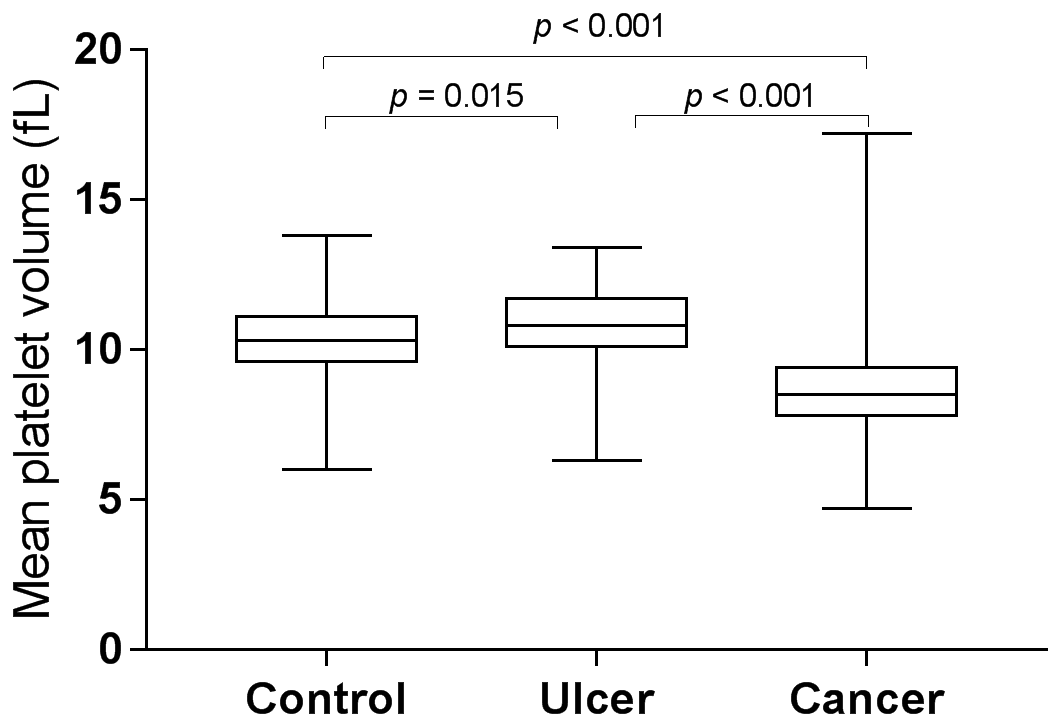
MPV levels in gastric cancer, gastric ulcer, and control group

**Figure 2:**
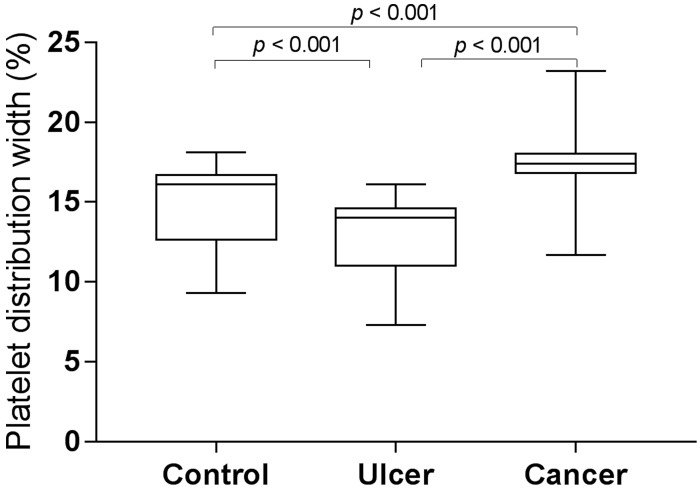
PDW levels in gastric cancer, gastric ulcer, and control group

MPV and PDW levels in gastric cancer, gastric ulcer, and control group are shown in Figure 1 and Figure 2. MPV levels were increased both in control group and in ulcer group compared to those in cancer group (control *vs*. cancer, *p* < 0.001; ulcer *vs*. cancer, *p* < 0.001, post hoc Tukey test). Furthermore, MPV levels of patients with gastric ulcer were higher compared to those of control subjects (*p* = 0.015, post hoc Tukey test). However, PDW levels were reduced both in control group and in ulcer group compared to those in cancer group (control *vs*. cancer, *p* < 0.001; ulcer *vs*. cancer, *p* < 0.001, post hoc Tukey test). Moreover, PDW levels of patients with gastric ulcer were lower compared to those of control subjects (*p* < 0.001, post hoc Tukey test).

Correlations between clinicopathological features and pre-operative MPV and PDW in gastric cancer are shown in Table 2. There were no significant differences in MPV and PDW among different serosa invasion, lymph node metastasis, distant metastasis, histological grade, and stage. PDW showed a difference in tumor size. However, MPV showed no difference in different tumor size group.

**Table 2:** Correlations between clinicopathological features and pre-operative MPV and PDW in gastric cancer.

Variables	N	MPV (fL)	P	PDW (%)	P
Serosa invasion (T stage)			0.853		0.745
T1+T2	61	8.7 (1.2)		17.4 (1.1)	
T3+T4	133	8.7 (1.5)		17.4 (1.2)	
Lymph node metastasis			0.958		0.285
Absence	62	8.7 (1.6)		17.5 (1.3)	
Presence	132	8.7 (1.3)		17.3 (1.0)	
Distant metastasis			0.113		0.289
Absence	187	8.7 (1.4)		17.4 (1.1)	
Presence	7	9.5 (1.4)		16.4 (2.3)	
Tumor size (cm)			0.367		0.048
<4.5	98	8.8 (1.6)		17.6 (1.2)	
=4.5	96	8.6 (1.2)		17.2 (1.1)	
Histological grade			0.234		0.152
Well/Moderately differentiated	34	8.5 (1.2)		17.1 (1.1)	
Low/Undifferentiated	160	8.8 (1.4)		17.4 (1.1)	
Stage			0.630		0.201
Ⅰ-Ⅱ	99	8.8 (1.5)		17.5 (1.2)	
Ⅲ-Ⅳ	95	8.7 (1.3)		17.3 (1.1)	

Values are shown as mean (standard deviation). MPV, mean platelet volume; PDW, platelet distribution width.

**Figure 3:**
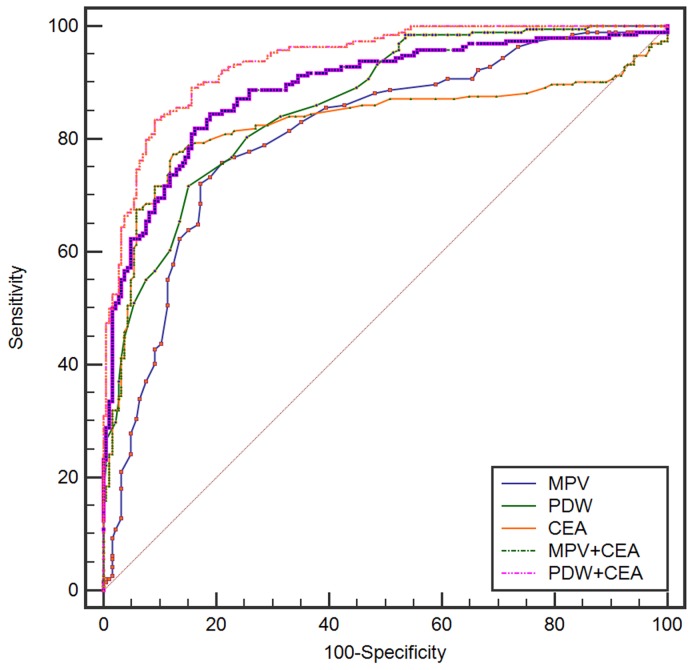
Receiver-Operator Characteristics (ROC) curve for MPV, PDW, and CEA combined showing sensitivity and 1-specificity of the differential diagnosis of gastric cancer *versus* controls

**Figure 4:**
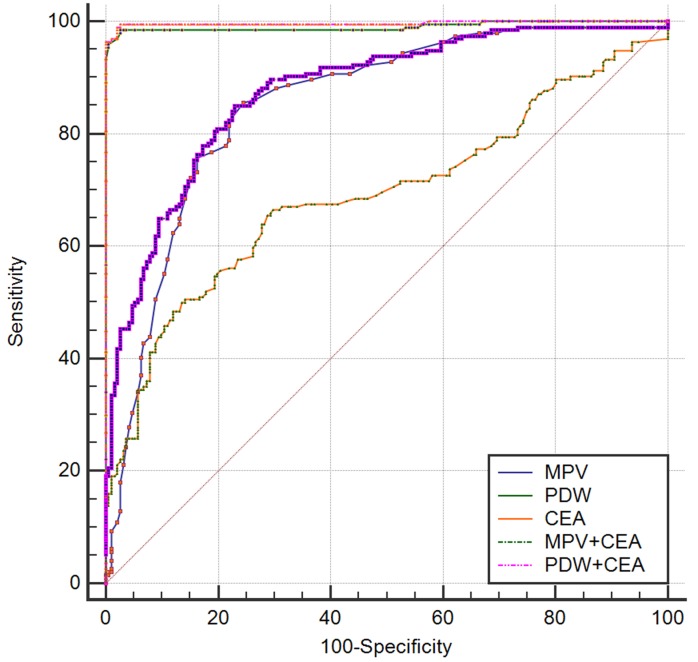
Receiver-Operator Characteristics (ROC) curve for MPV, PDW, and CEA combined showing sensitivity and 1-specificity of the differential diagnosis of gastric cancer *versus* gastric ulcer

ROC analysis was used to assess the AUC for single biomarkers and the combination of two (Table 3 and Table 4). When used to analyze control subjects *versus* gastric cancer, CEA, MPV, and PDW alone had the high specificity (82.7%-87.6%) and low sensitivity (71.7%-77.3%). The specificity elevated and sensitivity did not changed when the combination of MPV and CEA were applied. Moreover, the combination of PDW and CEA exhibited a significantly larger AUC of 0.939 (0.910-0.961) compared with the combination of MPV and CEA (*p* = 0.0045) (Figure 3). When used to analyze gastric ulcer *versus* gastric cancer, PDW alone had the high specificity (98.5%) and high sensitivity (97.4%). The specificity and sensitivity did not changed when the combination of PDW and CEA were applied. Moreover, PDW exhibited a significantly larger AUC of 0.996 (0.984-1.000) compared with the combination of MPV and CEA (*p* < 0.0001) (Figure 4).

**Table 3:** Receiver operating characteristic curve analyses showing the utility of alone or combined markers for differentiating of controls and gastric cancer.

Marker	Sensitivity	Specificity	PPV	NPV	AUC
CEA (ng/ml)	0.773	0.876	0.867	0.786	0.827 (0.785-0.863)
MPV (fL)	0.722	0.827	0.814	0.739	0.809 (0.766-0.848)
PDW (%)	0.717	0.849	0.832	0.741	0.863 (0.824-0.896)
CEA+MPV	0.820	0.838	0.841	0.816	0.889 (0.854-0.919)
CEA+PDW	0.835	0.908	0.905	0.840	0.939 (0.910-0.961)

Abbreviations: PPV, positive predictive value; NPV, negative predictive value; AUC, area under curve; CEA, carcinoembryonic antigen; MPV, mean platelet volume; PDW, platelet distribution width.

**Table 4:** Receiver operating characteristic curve analyses showing the utility of alone or combined markers for differentiating of gastric ulcer and gastric cancer.

Marker	Sensitivity	Specificity	PPV	NPV	AUC
CEA (ng/ml)	0.655	0.712	0.698	0.670	0.692 (0.643-0.737)
MPV (fL)	0.856	0.754	0.779	0.837	0.853 (0.813-0.887)
PDW (%)	0.985	0.974	0.974	0.984	0.990 (0.975-0.998)
CEA+MPV	0.851	0.770	0.789	0.835	0.876 (0.839-0.907)
CEA+PDW	0.990	0.979	0.980	0.989	0.996 (0.984-1.000)

Abbreviations: PPV, positive predictive value; NPV, negative predictive value; AUC, area under curve; CEA, carcinoembryonic antigen; MPV, mean platelet volume; PDW, platelet distribution width.

## DISCUSSION

In this study, we found that MPV levels are significantly increased and PDW levels are significantly reduced in patients with gastric ulcer and in control subjects compared with gastric cancer. Furthermore, combined detection of MPV, PDW and CEA is valuable in differentiating gastric cancer from gastric ulcer and controls.

Accumulating evidence revealed that platelet activation during cancer promotes disease progression. Several clinical studies have found the changed biomarkers of platelet activation, such as soluble P-selectin, CD40 ligand, and β-thromboglobulin in cancer [10-12]. Further study demonstrated that tumors could promote platelet production and activation by interleukin (IL)-6 pathway [13]. Consistent to previous findings, our study indirectly confirmed the results using a simple indicator of platelet activation. These data are also in line with the current knowledge that anti-platelet is considered to be a part of cancer adjuvant therapy [14].

The reasons for changes of MPV and PDW in gastric ulcer and gastric cancer are unclear. Bone marrow cells (including megakaryocytes) dys-regulation plays a key role. Platelet volume is determined both during megakaryopoiesis and during thrombopoiesis. Megakaryocytic maturation, platelet production and platelet size could be regulated by cytokines, such as IL-6, granulocytes colony stimulating factor (G-CSF) and macrophage colony stimulating factor (M-CSF) [15]. Considerable evidence suggests that IL-6 promotes tumorigenesis by regulating apoptosis, survival, angiogenesis, metastasis and metabolism [16]. In addition, megakaryopoiesis and subsequent thrombopoiesis in cancer may be stimulated by G-CSF and M-CSF, which could be secreted by tumor cells [17].

MPV was an early index of activated platelets. Increased MPV in gastric ulcer may be due to enhanced chronic inflammation and reduced MPV in gastric cancer was regarded as an increased consumption of large platelets [18]. Furthermore, MPV was positively associated with levels of thrombopoietin and IL-6, cytokines that regulate megakaryocyte ploidy [19, 20]. A report observed that MPV levels were increased in patients with gastric cancer compared with those in control subjects [21]. The result was not consistent to our findings. The discrepancies may be attributed to different sample sizes, dissimilar populations, and the variability of measurement methods. PDW is a measure of platelet heterogeneity. The heterogeneity in platelet volume is caused by heterogeneous demarcation of megakaryocytes [22]. However, the exact mechanism of changed PDW in gastric cancer still needs to be elucidated.

Compared to MPV, PDW or CEA alone, we found that PDW combined with CEA had high sensitivity and specificity. Therefore, it is helpful for early detection and early diagnosis of gastric cancer in asymptomatic patients or in patients with gastric ulcer.

Some limitations of the present study must be mentioned. First, the study was conducted in a single center. Second, a mechanistic explanation for our results is not provided by our data and further study is needed. Third, because the study includes only Chinese participants, the results cannot be generalized.

In conclusion, combined use of MPV, PDW and CEA can accurately distinguish gastric cancer from gastric ulcer and controls.

## MATERIALS AND METHODS

### Study population

The study involved 194 patients with gastric cancer (mean age 54.7 ± 9.7 years, range 28-77 years), 191 subjects with gastric ulcer (mean age 54.9 ± 5.2 years, range 35-71 years), and 185 control subjects (mean age 56.6 ± 3.7 years, range 50-72 years) from January 2014 to June 2014. The patients were recruited from clinic in the Third Affiliated Hospital and the control subjects were recruited from the check-up center in the Second Affiliated Hospital, Harbin Medical University. Control subjects were matched for age, gender, body mass index (BMI), and smoking status. Patients meeting all of the following requirements were included: (1) undergone complete surgical resection and diagnosis of gastric cancer was confirmed by histology; (2) untreated before diagnosis; (3) measurement of CEA before surgery. To be eligible, participants must not have reported any of the following: gastroduodenal diseases, hematological disorders, autoimmune diseases, systemic inflammatory diseases, coronary artery disease, hypertension, diabetes mellitus, thyroid disease, renal disease, hepatic disorder and other cancer, and medical treatment with anticoagulant, statins, and acetylic salicylic acid. Subjects gave written informed consent before the beginning of the study. The patients with gastric ulcer underwent upper digestive endoscopy with gastric biopsy. Tumors were classified according to the 7th edition of the AJCC/TNM tumor staging. The study protocol was approved by the Institutional Review Board of the Second and the Third Affiliated Hospital of Harbin Medical University.

### Clinical examination and biochemical measurements

All subjects completed a health-related questionnaire and underwent a detailed physical examination. Recorded information included smoking status, medical history and medication use for each subject. BMI was defined as weight divided by height in meters squared. A venous blood sample was collected from each participant under fasting conditions. The serum carcinoembryonic antigen (CEA) was assayed using an automatic electrochemistry luminescence immunoassay system (ROCHE cobas 8000; Roche, Germany). White blood cell (WBC), haemoglobin, and platelet indices were measured by an autoanalyzer (Sysmex XE-2100, Kobe, Japan). The whole blood samples were collected in EDTA-containing tubes, and all samples were processed within 30 minutes after blood collection. The inter- and intra-assays coefficients of variation (CVs) of all these assays were below 5%.

### Statistical analyses

The descriptive statistics are presented as means ± SD or medians (interquartile range) for continuous variables and percentages of the number for categorical variables. When baseline characteristics between two groups were compared, normally distributed continuous variables were compared with the Student t test and skewed-distributed with the Mann-Whitney U test. When baseline characteristics among three groups were compared, normally distributed continuous variables were compared with the one-way ANOVA and skewed-distributed with Kruskal-Wallis H test. The Chi-square test was used for categorical variables. Statistical analyses were carried out using SPSS Statistics version 22.0 (SPSS Inc., Chicago, IL, USA). Receiver-operating characteristic curves were used to define sensitivity and specificity, and the differences in the area under the curve (AUC) were detected by using MedCalc version 15.0. A *p* value < 0.05 was considered as statistically significant.
